# Association of rheumatoid factor and anti-cyclic citrullinated peptide positivity, but not carriage of shared epitope or *PTPN22* susceptibility variants, with anti-tumour necrosis factor response in rheumatoid arthritis

**DOI:** 10.1136/ard.2007.084715

**Published:** 2008-03-28

**Authors:** C Potter, K L Hyrich, A Tracey, M Lunt, D Plant, D P M Symmons, W Thomson, J Worthington, P Emery, A W Morgan, A G Wilson, J Isaacs, A Barton, BRAGGSS

**Affiliations:** 1 Arthritis Research Campaign Epidemiology Unit, University of Manchester, Manchester, UK; 2 Leeds Institute of Molecular Medicine, Section of Musculoskeletal Disease, University of Leeds, Leeds, UK; 3 Section of Musculoskeletal Sciences, University of Sheffield, Sheffield, UK; 4 Musculoskeletal Research Group, Institute of Cellular Medicine, Newcastle University, Newcastle-upon-Tyne, UK

## Abstract

**Objective:**

To determine whether rheumatoid factor (RF), anti-cyclic citrullinated peptide (CCP) antibodies, or carriage of shared epitope (SE) and *PTPN22* genetic susceptibility variants predict response to therapy in patients with rheumatoid arthritis (RA) treated with anti-tumour necrosis factor (TNF) agents.

**Methods:**

UK-wide multicentre collaborations were established to recruit a large cohort of patients treated with anti-TNF drugs for RA. Serum RF, anti-CCP antibody and SE status were determined using commercially available kits. *PTPN22* R620W genotyping was performed by Sequenom MassArray. Linear regression analyses were performed to investigate the role of these four factors in predicting response to treatment by 6 months, defined as the absolute change in 28-joint Disease Activity Score (DAS28).

**Results:**

Of the 642 patients analysed, 46% received infliximab, 43% etanercept and 11% adalimumab. In all, 89% and 82% of patients were RF and anti-CCP positive, respectively. Patients that were RF negative had a 0.48 (95% CI 0.08 to 0.87) greater mean improvement in DAS28 compared to patients that were RF positive. A better response was also seen among patients that were anti-CCP negative. No association was demonstrated between drug response and SE or *PTPN22* 620W carriage.

**Conclusion:**

The presence of RF or anti-CCP antibodies was associated with a reduced response to anti-TNF drugs. However, these antibodies only account for a small proportion of the variance in treatment response. It is likely that genetic factors will contribute to treatment response, but these do not include the well established RA susceptibility loci, SE and *PTPN22*.

To date, three anti-tumour necrosis factor (TNF) biological agents have been approved for the treatment of rheumatoid arthritis (RA), namely etanercept.^1^ Collectively these drugs have become one of the most effective methods of treating RA, with nearly half of all treated patients achieving an American College of Rheumatology 20% (ACR20) improvement level or higher. However, there is still a substantial proportion of patients who show partial or no response to anti-TNF therapy. With treatment limited by expensive annual costs in many countries, a number of studies have investigated predictors of response in treated patients.^2^^–^4 For example, analyses in a large-scale longitudinal observational study cohort identified lower baseline health assessment questionnaire (HAQ) scores and concurrent treatment with disease-modifying antirheumatic drugs (DMARDs) as predictors of greater response rates, although the latter was only significant in the group of patients treated with etanercept.^4^

Serological and genetic factors are also likely to contribute towards drug response. Several small studies (sample sizes <130) have investigated the utility of autoantibodies, including rheumatoid factor (RF) and anti-cyclic citrullinated peptide (anti-CCP), for predicting response to treatment with biological agents but results have been inconsistent.^2^ ^3^ ^5^ ^6^ Similarly, studies have investigated the role of genetic polymorphisms in the genes encoding TNF,^7^^–^12 interleukin (IL)1β and the IL1-receptor antagonist,^11^ ^13^ IL10,^13^ ^14^ transforming growth factor (TGF)β1^13^ and FcγRIIIA^15^ ^16^ with inconsistent findings. In particular, a significant association was demonstrated between carriage of the shared epitope (SE) and response to etanercept in a cohort of 200 patients in the USA^12^ but was not replicated in a smaller study of 123 patients from Sweden.^13^ Furthermore, no association of SE status with response to infliximab treatment was observed in two European populations (78 patients from Spain and 198 patients from France).^11^ ^17^ However, most of these studies were hindered by small sample sizes resulting in limited power to detect modest effects. In this study we established a UK-wide multicentre collaboration to recruit a large cohort of patients treated with anti-TNF agents and tested the hypothesis that confirmed RA susceptibility factors, namely RF, anti-CCP, SE and *PTPN22* 620W, are associated with clinical response in patients treated with anti-TNF.

## METHODS

### Patient selection

UK-wide multicentre collaborations were established to recruit patients treated with anti-TNF drugs for RA. Eligible patients from each centre were subsequently identified from the British Society of Rheumatology’s (BSR) Biologics Register (BR).^18^ This register compiles extensive clinical information on patients starting treatment with a biological agent and follows them prospectively, on a 6-monthly basis for 5 years, in order to monitor and determine the incidence of potential short and long term hazards. The following criteria were used for the selection of patients for the current study: (1) currently actively participating in the BSRBR long-term safety study, (2) doctor-confirmed diagnosis of RA, (3) currently or have been treated with one of the three anti-TNF biological agents, (4) European Caucasian descent and (5) reached 6 months of follow-up. Patients who stopped treatment temporarily during the first 6 months of therapy were excluded from selection. Similarly, patients who discontinued therapy prior to the 6-month follow-up for any reason other than inefficacy were excluded from selection.

### Patient recruitment and sample collection

Eligible patients from each collaborating centre were invited to take part in the study. Additional blood samples were obtained from consenting patients when they required a blood test as part of routine care. The additional blood samples and signed consent forms were posted to the Arthritis Research Campaign (arc) Epidemiology Unit for processing and storage. For the majority of patients, two samples of blood were taken: one for serum and one for DNA extraction. DNA was isolated using a standard phenol/chloroform extraction method. Serum and DNA samples were stored at −80°C. UK Central Office of Research Ethics Committees (COREC) approval (04/Q1403/37) was obtained for the study.

### Clinical information

Clinical and demographic data held on the BSRBR database was extracted, with the consultants’ permission, and compiled for each consenting patient. Disease activity was measured using the 28-joint count disease activity score (DAS28).^19^

### Immunogenetics

Serum RF and anti-CCP antibody titre were measured using commercially available kits (RF-PAIA Immunoturbidimetric Assay for rheumatoid factor, Diastat Anti-CCP Kit (Axis-Shield Diagnostics, Dundee, UK)). Patients with titres ⩾40 U/μl and ⩾5 U/μl were defined as positive for RF and anti-CCP antibodies, respectively. HLA-DRB1 typing was performed using commercially available kits (Dynal RELI SSO HLA-DRB1 Typing Kit (Dynal Biotech, Wirral, UK)). The SE was defined as the presence of any of the following alleles: human leukocyte antigen (HLA)-DRB1*0101, *0102, *0104, *0401, *0404, *0405, *0408 or *1001. In addition, *PTPN22* R620W (1858C/T) genotyping was performed using mass spectrometry (Sequenom, Cambridge, UK) as recommended by the manufacturer.

### Analysis

The primary outcome measure was absolute change in DAS28 between baseline and 6 months. Linear regression analyses were performed to investigate association between change in DAS28 and RF, anti-CCP status, SE and PTPN22*620W carriage. For the purposes of this analysis, the recorded 6-month DAS28 score was used whether patients had discontinued therapy or not. Analyses were adjusted for baseline DAS28, baseline HAQ score, administration of concurrent DMARDs and gender, as these factors have previously been shown to be significant independent predictors of response in the BSRBR cohort as a whole and were also associated in the current cohort (see supplementary material).^4^ Analyses were repeated excluding any patients with previous exposure to a biological drug, whether or not it was the same agent. In addition, interaction analyses were performed to determine whether any observed effects were similar across the two major drug types, namely etanercept and infliximab. Patients treated with adalimumab were excluded from this latter analysis due to the small numbers in this subgroup. Finally, the European League Against Rheumatism (EULAR) improvement criteria was assessed as a secondary outcome measure using logistic regression analyses and applying the same model as described above.^20^ Power calculations were performed using Quanto (http://hydra.usc.edu/gxe).^21^

## RESULTS

### Patient recruitment

Collaborations were established with 20 rheumatology centres across the UK from which 1485 patients receiving anti-TNF therapy for RA satisfied the study inclusion criteria. Of these, 1292 responded to the invitation letter (87%) with 1195 patients willing to take part (80%). DNA samples were extracted and available for the first set of 642 patients to be recruited, which were utilised in the current analysis.

### Baseline characteristics and immunogenetics

Baseline characteristics for the group of 642 patients are presented in table 1. Clinical and demographic measures were comparable to those previously reported across the BSRBR dataset as a whole, indicating that this cohort was representative of the larger anti-TNF-treated RA population in the UK.^22^

**Table 1:** Baseline characteristics

Baseline characteristics	Etanercept	Infliximab	Adalimumab	Combined
Number of cases	278 (43)	296 (46)	68 (11)	642
Age, years*	57 (11)	58 (11)	59 (12)	57 (11)
No. female	223 (80)	228 (77)	51 (75)	502 (78)
Current smokers	56 (20)	51 (17)	6 (9)	113 (18)
Ever smoked	163 (59)	168 (57)	35 (51)	366 (57)
Disease duration, years*	13 (9)	15 (10)	13 (10)	14 (10)
DAS28*	6.7 (1)	6.7 (1)	6.5 (1)	6.7 (1)
HAQ*	2 (0.6)	2 (0.6)	2 (0.5)	2 (0.6)
Concurrent DMARD(s)	152 (55)	277 (94)	38 (56)	467 (73)
Concurrent steroids	105 (38)	135 (46)	24 (35)	264 (41)
Previous biological	21 (8)	10 (3)	3 (4)	34 (5)

*Values are mean (SD). All other values are n (%).

DAS28, 28-joint disease activity score; DMARD, disease-modifying antirheumatic drug; HAQ, Health Assessment Questionnaire.

Genotyping of the *PTPN22* R620W (C1858T) polymorphism and SE was successfully performed in 96% and 83% of patients, respectively (table 2). Given the frequencies, there was more than 90% power to detect a difference of ⩾0.6 U in the absolute change in DAS28 following 6 months of therapy at the 5% significance level, for *PTPN22* and SE carriage in the current cohort. This level of improvement reflects the difference between non- and moderate-responders, based on the EULAR criteria. Autoantibody titres were available for 81% of patients (table 2), providing 77% and 91% power to detect the same effect described above for RF and anti-CCP positivity, respectively.

**Table 2:** Rheumatoid factor (RF), anti-cyclic citrullinated peptide (CCP), shared epitope (SE) and *PTPN22* status

	Etanercept	Infliximab	Adalimumab	Combined
RF positive	219/241 (91)	189/218 (87)	54/62 (87)	462/521 (89)
Anti-CCP positive	206/241 (86)	177/218 (81)	42/62 (68)	425/521 (82)
SE carriage	184/225 (82)	208/261 (80)	40/49 (82)	432/535 (81)
*PTPN22* carriage	78/268 (29)	93/287 (33)	17/64 (27)	188/619 (30)

Values are n of positive/total available (% positive).

### Predictors of response

By the first 6 months follow-up, 10% had discontinued treatment due to inefficacy while 90% continued anti-TNF therapy. Based on the EULAR improvement criteria, 21% of patients were non-responders, 52% moderate responders and 27% good responders. The mean change in DAS28 was an improvement of 2.5 points and this is consistent with data from the BSRBR as a whole.^22^ Baseline and absolute change in DAS28 values were normally distributed across the patient population.

Linear regression analyses were first performed to investigate association between drug response at 6 months, defined as the absolute change in DAS28, and the baseline factors listed in table 1. Of these, baseline DAS28, baseline HAQ score, concurrent DMARD therapy and gender were significantly associated with drug response (p⩽6.2×10^–3^; see supplementary material). These findings were expected as associations to these factors have been previously reported in the BSRBR data, from which the current cohort was recruited.^4^ Thus, in all analyses, adjustments were made for these baseline factors.

Linear regression analyses were subsequently performed to investigate association of drug response with RF, anti-CCP, SE and PTPN22*620W status (table 3). Compared to patients negative for RF, patients positive for RF demonstrated significantly less improvement in their DAS28 values following anti-TNF therapy (coefficient −0.48, 95% CI −0.87 to −0.08, p = 0.018) (table 3). Similarly, patients positive for anti-CCP antibodies demonstrated significantly less improvement in DAS28 compared to anti-CCP negative subjects (coefficient −0.39, 95% CI −0.71 to −0.07, p = 0.017) (table 3). Repeating the analysis after exclusion of patients with a previous exposure to a biological agent did not alter these conclusions (table 3). By contrast, the difference in anti-TNF treatment response between patients that were autoantibody positive and negative was not statistically significantly different when assessed using logistic regression analyses with the EULAR response criteria as the outcome measure, although a trend was observed (see supplementary material). This highlights the greater power of continuous compared to categorical data for such analyses. No association was demonstrated between drug response and either SE or PTPN22*620W carriage, under any model tested (p>0.05) (table 3 and supplementary material).

**Table 3:** Linear regression for rheumatoid factor (RF), anti-cyclic citrullinated peptide (CCP), shared epitope (SE) and *PTPN22*

Predictor	n* (%)	Mean DAS28* (SD)		Linear regression, coefficient (95% CI) p value	
		Base	Improvement	Adjusted 1†	Adjusted 2‡
RF negative	59 (11)	6.72 (1)	3.03 (1.7)	Ref	Ref
RF positive	462 (89)	6.59 (1)	2.43 (1.5)	−0.48 (−0.87 to −0.08) p = 0.02	−0.48 (−0.89 to −0.07) p = 0.02
Anti-CCP negatve	96 (18)	6.61 (1)	2.90 (1.6)	Ref	Ref
Anti-CCP positive	425 (82)	6.61 (1)	2.40 (1.5)	−0.39 (−0.71 to −0.07) p = 0.02	−0.39 (−0.72 to −0.06) p = 0.02
SE negative	103 (19)	6.65 (1)	2.38 (1.5)	Ref	Ref
SE positive	432 (81)	6.71 (1)	2.49 (1.5)	0.07 (−0.25 to 0.39) p = 0.68	0.06 (−0.26 to 0.39) p = 0.70
*PTPN22* negative	431 (70)	6.67 (1)	2.51 (1.6)	Ref	Ref
*PTPN22* positive	188 (30)	6.72 (1)	2.48 (1.4)	−0.11 (−0.36 to 0.15) p = 0.41	−0.13 (−0.39 to 0.13) p = 0.34

*Figures represent those across the complete subgroup of 642 patients. †Initial analyses were performed across the entire cohort, adjusting for baseline DAS28, HAQ, concurrent DMARD therapy and gender. ‡Subsequent analyses excluded patients with previous exposure to a biological agent.

DAS28, 28-joint Disease Activity Score; DMARD, disease-modifying antirheumatic drug; HAQ, Health Assessment Questionnaire; Ref, reference group.

The effects of RF and anti-CCP antibodies were investigated further by performing multivariate linear regression combining both antibodies, together with previously known predictors (baseline HAQ, concurrent DMARD therapy and gender). Being positive for RF and anti-CCP did not better predict response to anti-TNF therapy (RF only: R^2^ = 0.17, anti-CCP only: R^2^ = 0.17, RF plus anti-CCP: R^2^ = 0.17). Furthermore, there was no interaction between these two factors and their association with drug response (RF*anti-CCP: R^2^ = 0.18, p = 0.16). However, as the majority of patients were positive for both antibodies, these analyses may be underpowered.

Finally, in order to investigate whether the predictive effects of RF and anti-CCP antibodies were equal for etanercept and infliximab response, linear regression was performed including the interaction between drug type and autoantibody status. These analyses suggested that, although the effects of RF and anti-CCP antibodies appeared restricted to patients treated with infliximab, the effects were not statistically significantly different between the two major drug types (table 4).

**Table 4:** Linear regression of rheumatoid factor (RF) and anti-cyclic citrullinated peptide (CCP), stratifying for anti-tumour necrosis factor (TNF) agents

Predictor	n* (%)	Mean DAS28* (SD)		Linear regression coefficient (95% CI) p value	
		Base	Improvement	Adjusted†	Difference between drugs
Etanercept:					Ref
RF negative	22 (9)	6.63 (1)	2.69 (1.8)	Ref	
RF positive	219 (91)	6.63 (1)	2.43 (1.5)	−0.25 (−0.89 to 0.39) p = 0.44	
Infliximab:					−0.58 (−1.4 to 0.03) p = 0.18
RF negative	29 (13)	6.79 (1)	3.34 (1.6)	Ref	
RF positive	189 (87)	6.60 (1)	2.34 (1.6)	−0.83 (−1.40 to −0.27) p = 0.004	
Etanercept:					Ref
Anti-CCP negative	35 (15)	6.62 (1)	2.72 (1.4)	Ref	
Anti-CCP positive	206 (85)	6.64 (1)	2.40 (1.5)	−0.26 (−0.77 to 0.26) p = 0.33	
Infliximab:					−0.41 (−1.1 to 0.3) p = 0.26
Anti-CCP negative	41 (19)	6.62 (1)	3.07 (1.7)	Ref	
Anti-CCP positive	177 (81)	6.63 (1)	2.33 (1.6)	−0.67 (−1.16 to −0.18) p = 0.007	

*Figures represent those across the complete subgroup of 642 patients. †Analyses adjusted for baseline DAS28, HAQ, concurrent DMARD therapy and gender.

DAS28, 28-joint Disease Activity Score; DMARD, disease-modifying antirheumatic drug; HAQ, Health Assessment Questionnaire; Ref, reference group.

## DISCUSSION

The introduction of anti-TNF biological agents has transformed the management of RA. However, a substantial proportion of treated patients still demonstrate partial or no response to these therapies. Previous studies have suggested that the effect of clinical factors alone in predicting response is relatively modest.^2^^–^4 Hence, in the current study, we have focused on genetic and serological markers. In keeping with previous reports, we have shown that the presence of RF and anti-CCP antibodies is associated with a significantly reduced improvement in the DAS28 score following 6 months of anti-TNF therapy. No associations were demonstrated between drug response and carriage of risk alleles for either of the other two well established RA susceptibility factors, SE or *PTPN22*.

There are a number of methodological limitations to the study, which require discussion. Firstly, although the current study design may inform predictions of how patients receiving anti-TNF therapies will respond to those treatments, the lack of a control group of patients with RA that were not anti-TNF treated means that the study cannot inform the debate about whether a patient will respond better to therapy with an anti-TNF rather than a DMARD treatment.

Secondly, response measures were assessed at 6 rather than 3 months, when clinical decisions regarding the continuation of therapy are usually made. Consequently, ∼10% of patients had discontinued therapy due to inefficacy prior to the 6-month follow-up and some will have commenced alternative treatment to which they may have responded. Hence, the DAS28 at 6 months may not be a true reflection of the DAS28 when the drug was discontinued. However, this subgroup of patients generally remained non-responders at 6 months despite possibly receiving alternative drugs (mean DAS28 improvement at 6 months: 0.8 compared to 2.7 across the remainder of the cohort). As the study aims to identify predictors of response by 6 months rather than predictors of response only in those who remain on treatment, these patients were included in the analysis although we recognise that this may have resulted in underestimations of observed effects.

Thirdly, as one of the requirements for prescribing anti-TNF agents in the UK includes failure of at least two previous DMARDs, the patients recruited have severe, long-standing RA with a mean duration of 14 years. As discussed by Hyrich *et al*, patients with more severe disease as a result of irreversible joint damage may be less likely to respond to treatment.^4^ In order to account for this, analyses were repeated adjusting for disease duration, but this did not change the overall conclusions (see supplementary material).

Lastly, the serology was measured cross-sectionally at the time of sample collection, which may have been some time after commencement of treatment. Previous studies have shown that, although titres are affected by treatment, status generally is not.^6^ ^23^ Based on records held on the BSRBR, antibody status changed from positive to negative during anti-TNF treatment in less than 3% of patients included in the current analyses. Hence, in all the analyses, autoantibody status rather than titre has been used. It should also be noted that the proportion of patients that were RF positive in the current study is higher than that reported previously for the BSRBR cohort as a whole (89% vs 72%, respectively).^4^ This is most likely to be due to differences in data collection methods: the BSRBR study relies on information being provided by the contributing doctors whereas, for the purposes of the current study, RF was remeasured in all patients for whom a serum sample was available.

Conversely, our study has several advantages over previous investigations. Importantly, the use of the BSRBR to identify suitable patients has meant that the subgroup studied is comparable to the BSRBR in its entirety. As, until relatively recently, almost all patients receiving an anti-TNF drug in the UK for RA were included on this register, the cohort studied is likely to reflect the characteristics of patients treated with anti-TNF as a whole, at least in the UK. Furthermore, a wealth of clinical and demographic data had already been collected, creating a well characterised cohort. In addition, this is a large cohort, allowing robust inferences to be drawn. Finally, the use of the DAS28 measure rather than the EULAR response criteria enhances the power of the study to detect association with genetic predictors of response in patients with RA treated with anti-TNF.

As no correction for multiple testing was applied in the current analyses, these results will require validation in similar-sized cohorts. Nonetheless, the findings support those of smaller studies in which similar trends between drug response and baseline RF and anti-CCP antibody titres have been demonstrated.^2^ ^3^ ^6^ As discussed by Mewar *et al*, RF and anti-CCP antibodies are independent markers of disease severity for RA.^24^ Thus the present findings could be interpreted as showing that those patients with the most severe disease are least likely to respond to these therapies. Indeed, there is some evidence to support this hypothesis, as HAQ score, a measure of disability, was also significantly associated with response. However, RF and anti-CCP antibody status remained significant predictors of response even after accounting for markers of severity such as HAQ and disease duration.

It is salient to note that combining information on clinical markers of anti-TNF treatment response previously identified, (concurrent DMARD therapy, baseline HAQ and gender)^2^^–^4 with RF and anti-CCP antibody status data still only accounts for a small proportion of the variance in drug response (R^2^ = 17%) and would not be useful in the clinical setting. We hypothesise that, in addition to these clinical and serological factors, genetic factors will play a role and the challenge now is to identify these. No association was observed between treatment response and carriage of the RA susceptibility allele of the *PTPN22* gene and, in keeping with most previous studies, no association of treatment response was observed with SE carriage, a well established RA severity and susceptibility locus. Several novel RA susceptibility loci have recently been reported (eg, *OLIG3/TNFAIP3*, *TRAF1/C5* and *STAT4*), which may also warrant investigation.^25^^–^28 However, genes contributing to disease susceptibility may be different to those that determine response to treatment.

In summary, the presence of RF or anti-CCP antibodies was associated with a reduced response to anti-TNF drugs in patients with RA treated with anti-TNF. However, the presence of these antibodies only accounts for a small proportion of the variance in treatment response. It is likely that genetic factors will contribute to determining the response to treatment with these agents but do not include the well established RA susceptibility loci, SE or *PTPN22*.

## APPENDIX

### Members of the Biologics in Rheumatoid Arthritis Genetics and Genomics Study Syndicate (BRAGGSS)

Cambridge University Hospitals NHS Foundation Trust (Dr A J Crisp, Professor J S H Gaston, Dr F C Hall, Dr B L Hazleman, Dr J R Jenner, Dr A Ostor, Dr B Silverman, Dr C Speed).

County Durham and Darlington Acute Hospitals NHS Trust (Dr D. Armstrong, Dr A J Chuck, Dr S Hailwood).

Derby Hospitals NHS Foundation Trust (Dr L J Badcock, Dr C M Deighton, Dr S C O’Reilly, Dr M R Regan, Dr Snaith, Dr G D Summers, Dr R A Williams).

Doncaster And Bassetlaw Hospitals NHS Foundation Trust (Dr J R Lambert, Dr R Stevens, Dr C Wilkinson).

Gateshead Health NHS Trust (Dr J Hamilton, Dr C R Heycock, Dr C A Kelly, Dr V Saravanan).

Hereford Hospitals NHS Trust (Dr D H Rees, Dr R B Williams).

Mid Staffordshire General Hospitals NHS Trust (Dr S V Chalam, Dr D Mulherin, Dr T Price, Dr T Sheeran).

Morecambe Bay Hospitals NHS Trust (Dr M Bukhari, Dr W N Dodds, Dr J P Halsey).

Norfolk and Norwich University Hospital NHS Trust (Dr K Gaffney, Professor A J Macgregor, Dr T Marshall, Dr P Merry, Professor D G I Scott).

Pennine Acute Hospitals NHS Trust (Dr B Harrison, Dr M Pattrick, Dr H N Snowden).

Peterborough and Stamford Hospitals NHS Foundation Trust (Dr N J Sheehan, Dr N E Williams).

Portsmouth Hospitals NHS Trust (Dr R G Hull, Dr J M Ledingham, Dr F Mccrae, Dr M R Shaban, Dr A L Thomas).

Sandwell and West Birmingham Hospitals NHS Trust (Prof C D Buckley, Dr D C Carruthers, Dr R Elamanchi, Dr P C Gordon, Dr K A Grindulis, Dr F Khattak, Dr K Raza, Dr D.Situnayake).

Sheffield Teaching Hospitals NHS Trust (Dr M Akil, Dr R Amos, Dr D E Bax, Dr S Till, Dr G Wilson, Dr J Winfield).

South Tees Hospitals NHS Trust (Dr F Clarke, Dr J N Fordham, Dr M J Plant, Dr Tuck).

St Helens and Knowsley Hospitals NHS Trust (Dr V E Abernethy, Dr J K Dawson, Dr M Lynch).

The Leeds Teaching Hospitals NHS Trust (Dr S Bingham, Professor P Emery, Dr A Morgan).

The Newcastle upon Tyne Hospitals NHS Trust (Dr F Birrell, Mr P Crook, Dr H E Foster, Dr B Griffiths, Dr I D Griffiths, Dr M L Grove, Professor J D Isaacs, DR L Kay, Dr A Myers, Dr P N Platt, Dr D J Walker).

University Hospital Birmingham NHS Foundation Trust (Dr Bowman, Dr P Jobanputra, Dr R W Jubb, Dr E C Rankin).

University Hospital of North Staffordshire NHS Trust (Dr E H Carpenter, Dr P T Dawes, Dr A Hassell, Professor E M Hay, Dr S Kamath, Dr J Packham, Dr M F Shadforth).

Whipps Cross University Hospital NHS Trust (Dr S P Donnelly, Dr D Doyle, Dr A Hakim, Dr J G Lanham).
